# Recruiting children and young people with vision impairment for clinical research – experience from the SeeMyLife study

**DOI:** 10.1186/s12874-026-02915-z

**Published:** 2026-06-16

**Authors:** Lisa Gittel, Robert P. Finger, Verena Richter, Asmaa Cadi, Nicole Beck, Alice Colombo, Arvydas Gelžinis, Erika Gustafierro, Francesca Incagli, Agnė Kručaitė, Martina Lanza, Matilde Leonardi, Bart P. Leroy, Dominika Nowakowska, Katarzyna Nowomiejska, Francesco Parmeggiani, Valérie Pelletier, Anais Philippe, Maria Eleonora Reffo, Yaël Slaghmuylder, Agnese Suppiej, Chloé Laeng, Reda Žemaitienė, Hélène Dollfus, Jan H. Terheyden, SeeMyLife consortium members, J. Birtel, R. Butkeviciene, L. Danuseviciene, V. Gouriant, E. Guastafierro, G. Gwizda, E. Lauwerier, D. LeBreton, A. Lomo, B. Prinz, Jugnoo S. Rahi, M. Samardakiewicz, D. Yang

**Affiliations:** 1 Department of Ophthalmology, University Hospital Bonn, Bonn, Germany; 2 Department of Ophthalmology, Medical Faculty Mannheim, University of Heidelberg, Mannheim, Germany; 3 Hôpitaux universitaires de Strasbourg, Strasbourg, France; 4 Lithuanian University of Health Sciences, Kaunas, Lithuania; 5 SC Neurologia, Salute Pubblica, Disabilità, Fondazione IRCCS Istituto Neurologico Carlo Besta, Milano, Italy; 6 Department of Head and Skin, Ghent University, Ghent, Belgium; 7 Department of Ophthalmology & Center for Medical Genetics, Ghent University Hospital, Ghent, Belgium; 8 Medical University of Lublin, Lublin, Poland; 9 Department of Translational Medicine and for Romagna, University of Ferrara, Ferrara, Italy; 10 Center for Retinitis Pigmentosa of Veneto Region, Camposampiero Hospital, Azienda ULSS 6 Euganea, Padova, Italy; 11 Robert Hollman Foundation, Padova, Italy; 12 Pediatric Unit, Department of Medical Sciences, University of Ferrara, Ferrara, Italy

**Keywords:** Recruitment challenges, Recruitment strategies, Clinical trials, Disability, Pediatrics, Visual impairment

## Abstract

**Background:**

Effective participant recruitment is crucial for the success of clinical trials, yet little is known about facilitators and barriers to recruitment in pediatric populations with visual impairment. Understanding which methods yield the highest participant engagement can help optimize recruitment efforts and improve study outcomes. Thus, we assessed factors associated with recruitment in the SeeMyLife study, a European multi-center cohort study about quality of life and participation of children and young people with visual impairment.

**Methods:**

Data on the perceived effectiveness of recruitment strategies were collected from principal investigators, study coordinators, clinical and research staff using an online survey. Additional information on recruitment approaches was obtained through document analysis of study-related communications, including e-mails and meeting minutes. Descriptive statistics were used to summarize recruitment rates and perceived effectiveness scores. Open-ended responses regarding recruitment challenges were analyzed thematically to identify common barriers and facilitators.

**Results:**

Seventeen clinical and research staff members from six countries participated in the survey and shared their insights and experience with participant recruitment. In total, study staff reported using twelve distinct recruitment sources to enhance participant enrolment. These sources were grouped into three broad categories: healthcare settings, community engagement and educational settings, and professional and system-level approaches. The recruitment of participants using the hospital information system was perceived as the most effective recruitment strategy, used by 71% of respondents. Other highly ranked sources included pediatric visual rehabilitation centers, patient associations, and affiliated university ophthalmology departments, where staff actively presented the study to their patients and referred eligible participants. Commonly reported recruitment challenges included restrictive inclusion criteria, limited parental responsiveness, concerns about the impact of study participation on children’s well-being, and institutional barriers.

**Conclusions:**

Successful strategies for enrolling children and young people with visual impairment into clinical research include direct interactions between clinical staff and families, likely due to established trust, easier identification of eligible participants, and clearer communication about the study. In contrast, recruitment approaches outside clinical care settings were perceived as less effective. These findings highlight the importance of integrating recruitment efforts within established healthcare networks when conducting pediatric vision impairment research.

**Supplementary Information:**

The online version contains supplementary material available at 10.1186/s12874-026-02915-z.

## Background

Between 11% and 40% of pediatric studies are terminated early due to challenges in recruitment, with low participant accrual presenting a major risk factor of early termination [1, 2]. This poses a significant challenge on the implementation of good clinical practice guidelines and ethical conduct of clinical research [3] and is echoed by the causes of trial failure in adult populations, where a low recruitment rate is the main cause of early termination [4, 5]. Despite the importance of robust recruitment strategies for the planning of clinical trials in children and young people and for the trials’ success and validity of results [6], the available research on recruitment barriers in pediatric patient populations is currently limited.

Previous research suggests that parental concerns (e.g., potential risk to the child), burdens of participation and lack of trust may play a more pivotal role during the recruitment of child and adolescent populations compared to adults [7, 8]. According to Kling et al. (2021), common obstacles include the fear of disclosing personal information and psychosocial stressors (e.g., being reminded of difficulties). Parents often perceive research topics as sensitive, making them hesitant to allow participation [9]. Additional practical constraints explaining low recruitment rates in pediatric studies include limited availability of children due to school obligations and difficulties faced by working parents in taking time off [10].

While effective trial recruitment strategies for general pediatric populations have been a focus of research [9, 10], it is largely unclear which factors influence the successful recruitment of children and young people with disabilities. In the European multi-center study SeeMyLife, we recruit children and young people with visual impairment (VI) due to rare eye conditions across six countries for cross-cultural validation of patient-centered trial outcomes and to better understand unmet needs in this patient population. In the SeeMyLife study, we have gained experience with facilitators that could support recruitment of other trials. We have therefore explored which participant recruitment strategies were deemed most effective according to the perspectives of the study personnel, while also identifying key challenges encountered during recruitment.

## Methods

To gain insights into the recruitment process for the SeeMyLife study and to identify which recruitment methods were most effective in engaging children and young people with VI, we explored the various recruitment approaches used and their perceived effectiveness according to study personnel. The evaluation included a systematic document analysis of communications (including e-mails and conference minutes) to identify strategies used and a brief online survey, in which we asked staff across the multiple study sites to rate the effectiveness of recruitment sources in the SeeMyLife study based on their experience. Only study personnel directly involved with the SeeMyLife study were included. As our study did not involve patients, institutional review board approval was not required. Our study followed the tenets of the declaration of Helsinki.

### The SeeMyLife study

SeeMyLife is a European multi-center non-interventional cohort study focusing on assessing the quality of life and participation of children and young people (aged 8–17 years) with VI. The main study objective is the cross-cultural validation of patient-reported candidate endpoints for children and young people with VI to use in clinical trials and to ultimately validate novel treatment strategies. This work builds on previous project-related research demonstrating a clear need for integrated, multidimensional assessment programs that evaluate the impact of visual impairment on quality of life, functioning, and participation in this population, as VI has consistently been shown to negatively affect these dimensions [11]. The participating countries in the SeeMyLife study are Belgium, France, Germany, Italy, Lithuania and Poland. The study team involves researchers with multidisciplinary backgrounds and clinicians from medicine, social sciences and psychology. SeeMyLife consists of quantitative, qualitative and mixed methods parts. The quantitative part includes up to 900 children and young people and their caregivers and collects sociodemographic, visual function and patient-reported outcome (PRO) data, using the Functional Vision Questionnaire (FVQ), the Vision-Related Quality of Life Questionnaire (VQoL), and the Participation and Environment Measure for Children and Youth (PEM-CY) [12–14]. The qualitative component includes follow-up interviews with a subgroup of participants, their caregivers, and healthcare professionals to gain deeper insight into the experiences and needs of children and young people with VI. The mixed-methods component integrates findings from both study parts.

### Analysis

Identification of the recruitment approaches used was based on a content analysis of relevant documents. Analyzed study documents were shared within the SeeMyLife consortium and included written e-mail communication and conference minutes, alongside informal notes of project management staff on study recruitment measures. A qualitative researcher extracted individual recruitment measures from these sources based on a coding strategy that was successively developed for the study. Individual screening sources were then categorized on a content basis. The individual screening sources identified were then embedded in an anonymized online survey (provided in Additional File 1). Collected data included anticipated and actual weekly recruitment rates; identified recruitment challenges (open-ended responses); recruitment sources utilized (multiple choice); perceived effectiveness of each recruitment source (Likert scale: 0–10). We used descriptive statistics to summarize recruitment rates and perceived effectiveness. Statistical analysis was performed using IBM SPSS, versions 25 (IBM, Armonk, NY) and *p* values < 0.05 were considered statistically significant. The open-ended responses regarding recruitment challenges were analyzed using thematic analysis following the approach described by Braun and Clarke (2006) to identify recurring themes and patterns in the data [15].

## Results

The content analysis of recruitment sources yielded 12 distinct recruitment sources that could be classified as direct care settings; community engagement and educational services; or professional and system-level approaches. A total of 17 clinical and research staff from six countries participated in the survey. Eight research assistants, five principal investigators, three study coordinators and one clinician provided insights into their recruitment efforts. Before the recruitment process began, the anticipated participation rate for the SeeMyLife study was 9.4 ± 8.7 participants per month. The actual recruitment data revealed a noticeably lower participation rate, averaging 5.1 ± 3.4 participants per month (−4.3 ± 5.3, p < 0.001).

The recruitment sources identified during content analysis were aligned with the results of the free-text data obtained from the survey, following three broader categories (Table 1). The first category, direct care settings, included recruitment through hospital information systems and healthcare or rehabilitation environments where children and young people with VI receive direct medical care and where staff actively presented the study to their patients and referred eligible participants. The second, community engagement and educational services, encompassed educational institutions and organizations providing developmental support. The third, professional and system-level approaches, referred to recruitment efforts initiated through professional networks or policy-level mechanisms.

**Table 1:** Overview of recruitment sources and their broader categories

Categories	Recruitment source
Direct care settings	Hospital information system Affiliated university ophthalmology departments Affiliated ophthalmology practices Pediatric visual rehabilitation centers Opticians and optical specialty stores
Community engagement and educational services	Specialized schools for students with VI Support groups for visually impaired and blind children and young people Leisure activity centers for individuals with VI Associations for individuals with VI
Professional and system-level approaches	Reimbursement* Pharmacies Promoting the study at a conference**

^*^The German research team provided financial compensation for study participation. Participants received 25 euros for completing the quantitative component of the study and additional 25 euros for participating in the qualitative component, which involved more extensive follow-up interviews

^**^The Lithuanian research team promoted study participation by presenting the study at the Lithuanian Society of Ophthalmology Annual Conference, as the conference provided direct access to ophthalmologists and other healthcare professionals involved in the care of children and young people with VI

The three broader categories and all 12 individual recruitment strategies were systematically assessed for their effectiveness using a scale from 0 (ineffective) to 10 (highly effective) in the survey. Among the broader categories, recruitment through direct care settings was the most frequently used strategy, with a total of 40 mentions. Of the 17 study personnel who completed the survey, 12 reported recruiting via hospital information system, 6 through affiliated university ophthalmology departments, 7 through affiliated ophthalmology practices, 11 through pediatric visual rehabilitation centers, and 4 through opticians and optical specialty stores, resulting in 40 total mentions within this category. Recruitment through direct care settings received an average perceived effectiveness score of 5.2 ± 3.0. This was followed by community engagement and educational services (32 uses, effectiveness 3.7 ± 2.9) and professional and system-level approaches (3 uses, effectiveness 3.3 ± 2.5). Within these broader categories, the hospital information system emerged as the most successful method, being utilized 12 times and achieving an average effectiveness score of 6.7 ± 2.5. Other highly ranked strategies included pediatric visual rehabilitation centers (11 uses, effectiveness 5.5 ± 3.6), associations for individuals with VI (13 uses, effectiveness 4.9 ± 3.1), affiliated university ophthalmology departments (6 uses, effectiveness 4.8 ± 2.7) and other sources (2 uses, effectiveness 4.5 ± 2.1), namely the introduction of a participant reimbursement and the presentation of the study at a conference. Additional methods, such as affiliated ophthalmology practices (7 uses, 4.4 ± 2.5), specialized schools for students with VI (8 uses, 3.8 ± 3.4) and support groups for visually impaired and blind children and young people (7 uses, 2.6 ± 1.3), demonstrated moderate perceived effectiveness. Meanwhile, opticians and optical specialty stores (4 uses, 1.5 ± 1.0), leisure activity centers for individuals with VI (4 uses, 1.3 ± 0.5) and pharmacies (1 use, 1.0) were rated the least effective recruitment channels.

Thirteen respondents mentioned having encountered recruitment challenges and reported them in the open-ended questions of the survey. The thematic analysis revealed that the most frequent challenges in pediatric participant recruitment can be grouped into three key themes: restrictive inclusion criteria (mentioned eight times), parental and participant engagement issues (mentioned five times), and institutional barriers (mentioned four times). Families’ responsiveness emerged as a recurring concern, with study staff noting that parents who initially consented often became unresponsive to follow-up requests, such as questionnaire completion. In the open-ended responses, study personnel explained that some parents expressed concerns about the potential impact of study participation on their child’s well-being. This may partly explain the lower participation rate than anticipated and further underscores the central role of parental decision-making in the pediatric recruitment process.

## Discussion

Recruitment remains one of the most significant challenges in clinical and research studies, particularly those involving pediatric populations. High rates of early study termination, up to 40%, are often attributed to slow or insufficient participant accrual [1, 2]. While enrolling children is not an independent predictor of recruitment failure, additional barriers, such as parental consents, emotional sensitivities, and logistical constraints pose significant challenges to study participation [7–10, 16]. These challenges are further amplified in studies involving children and young people with disabilities [17], a finding that was also reflected in our study. However, targeted strategies such as personalized outreach, involvement of trusted clinical staff and clear communication have shown to positively influence recruitment outcomes [9, 10, 18], which is echoed by our study’s main finding that recruiting children and young people in direct care settings is most promising. Hence, we particularly recommend this strategy for recruiting impaired children and young people.

With this study, we have explored clinical trial recruitment sources for pediatric population with VI. Three categories of recruitment sources emerged, which can be summarized as direct care settings, community engagement and educational services, professional and system-level approaches.

Recruiting participants directly at the hospital via the hospital information system was ranked as the most effective strategy. This in-house strategy likely benefits from direct access to patient records and streamlined identification of eligible participants. Rehabilitation centers, patient associations, affiliated university hospitals and other sources were also suggested to be effective recruitment sources. These methods may have been effective due to their established networks and engagement with specific patient populations. A key common factor among these methods is direct patient contact, which previous reviews have identified as a critical facilitator of pediatric research participation. Further, an established foundation of relationship and trust whether in the recruiting researcher or the institution itself can substantially facilitate enrolment, as demonstrated by previous research and supported by our findings in the SeeMyLife study [8, 19–22].

Several other strategies were found to be notably less effective according to our study personnel. Recruitment through affiliated ophthalmology practices, specialized schools and support groups showed varying degrees of effectiveness. Their more targeted but often smaller participant pools may explain this variability. Recruitment efforts through opticians, leisure centers and pharmacies yielded the lowest effectiveness scores, suggesting that these channels had limited reach, did not align well with the study's target population, or did not allow for the provision of sufficiently clear and comprehensive study information. Unlike hospital-based recruitment, no study personnel were present at these sites to provide additional information or answer questions. This aligns with previous findings indicating that a lack of understanding regarding study details is a frequently reported barrier to participation [8]. Moreover, parents might grant more authority to the treating hospital staff in comparison with other not directly involved professionals or associations.

Our findings highlight three interrelated challenges to pediatric recruitment: restrictive inclusion criteria, difficulties engaging parents and participants, and institutional barriers. Particularly notable was the issue of parental responsiveness. In the open-ended questions about recruitment challenges study staff reported that families who initially agreed to participate later became unresponsive (e.g., not completing questionnaires or answering follow-ups). As parents act as gatekeepers to study enrolment, their concern that participation imposes potential burden on their child is a critical factor influencing their consent decisions. These results align with previous research emphasizing the central role of parental attitudes and suggest that strategies to support parental reassurance and sustained engagement are essential for improving pediatric trial enrollment [7–9, 20].

Because such concerns can be discussed and sufficient information about the study can be provided directly during clinic visits, recruitment through direct care settings may help to overcome these barriers. Addressing these challenges through enhanced communication strategies and stronger collaborations with institutions and support networks may improve recruitment outcomes in future studies.

Strengths of our study include its international setting with six European countries participating to its results and broad experience with multi-center studies of the study staff that allowed us to extrapolate the results and draw conclusions beyond the SeeMyLife study. However, we acknowledge the possibly limited external validity of our results given the size of our sample. Furthermore, our study is limited by the fact that barriers to participation were assessed solely from the perspective of study staff. Future research should also investigate the perspectives of children and their families regarding participation in research studies, as these may provide additional insights into factors influencing recruitment and engagement.

## Conclusions

The results of our analysis highlight the importance of recruitment strategies embedded within direct care settings for enrolling children and young people with visual impairment into clinical research. Recruitment through hospital information system was perceived as the most effective approach for identifying and enrolling eligible participants, followed by pediatric visual rehabilitation centers and affiliated ophthalmology departments. Recruitment approaches outside established healthcare structures were perceived as less effective. Reported barriers included restrictive inclusion criteria, limited parental responsiveness, and institutional challenges, emphasizing the need for targeted communication strategies and strong collaboration with clinical partners. To optimize future recruitment efforts, researchers should consider leveraging hospital-based recruitment strategies while addressing key barriers through targeted communication strategies and stronger institutional collaborations.

## Supplementary Information

Supplementary Material 1.

## Data Availability

The data that support the findings of this study are available from the corresponding author on reasonable request.

## Abbreviations

FVQ: Functional Vision Questionnaire
ISPOR: International Society for Pharmacoeconomics and Outcomes Research
PEM-CY: Participation and Environment Measure – Children and Youth
PRO: Patient-reported outcome
VI: Visual impairment
VQoL: Vision-Related Quality of Life Questionnaire
