# Supplementary material for: Recruiting children and young people with vision impairment for clinical research – experience from the SeeMyLife study
Source: BMC Med Res Methodol. 2026 Jun 16;26:138. doi: 10.1186/s12874-026-02915-z (PMC13270738; doi:10.1186/s12874-026-02915-z)
Supplement: Supplementary file 1 — Supplementary Material 1. [file 12874_2026_2915_MOESM1_ESM.docx]

Dear SeeMyLife study teams, 

as indicated during our weekly meeting, we would like to invite you to participate in a short survey (estimated 3-5 min.) on effective recruitment strategies in the quantitative part of the SeeMyLife study. The goal of this is to share our experience in a scientific publication based on the results involving you.

We kindly invite all clinical and research staff involved with SeeMyLife to take part.

Please reply before March 10 so we can assemble and share the results of the survey during our next virtual call.

Thank you in advance,
Robert Finger and Jan Terheyden

1. Which consortium member country are you based in?
   - Belgium
   - France
   - Germany
   - Italy
   - Lithuania
   - Poland
2. What is your role in the SeeMyLife study?

- Principle investigator
- Study coordinator
- Research assistant
- Study nurse
- Clinician

1. Before the start of the SeeMyLife recruitment, how many participants did you expect to recruit per month on average? Please provide an estimated number (quantitative part).
2. On average, how many participants are recruited per month at your study center? Please provide an estimated number (quantitative part).
3. Which challenges have you encountered during patient recruitment for the SeeMyLife study? Please describe the main challenges at your study site in short keywords. You can use bullet points to structure your response.
4. Which sources of recruitment have been used at your study center? Please select all that apply.

- Hospital information system
- Specialized schools for students with visual impairment
- Associations for individuals with visual impairment
- Support groups for visually impaired and blind children and young people
- Opticians and optical specialty stores
- Rehabilitation centers for children and young people with visual impairment
- Affiliated ophthalmology practices
- Affiliated university eye hospitals
- Pharmacies offering specialized services for visually impaired patients
- Leisure activity centers for individuals with visual impairment
- Others – please specify

1. Please rank the effectiveness of the recruitment strategies used on a scale from 0 to 10 (0 = ineffective, 10 = highly effective).
